# How delayed and non-adherent treatment contribute to onward transmission of malaria: a modelling study

**DOI:** 10.1136/bmjgh-2019-001856

**Published:** 2019-12-10

**Authors:** Joseph D Challenger, Bronner P Gonçalves, John Bradley, Katia Bruxvoort, Alfred B Tiono, Chris Drakeley, Teun Bousema, Azra C Ghani, Lucy C Okell

**Affiliations:** 1 MRC Centre for Global Infectious Disease Analysis, Department of Infectious Disease Epidemiology, Imperial College London, London, United Kingdom; 2 Department of Immunology and Infection, London School of Hygiene and Tropical Medicine, London, United Kingdom; 3 MRC Tropical Epidemiology Group, London School of Hygiene and Tropical Medicine, London, UK; 4 Department of Research and Evaluation, Kaiser Permanente Southern California, Pasadena, California, USA; 5 Department of Global Health and Development, London School of Hygiene and Tropical Medicine, London, United Kingdom; 6 Public Health Department, Centre National de Recherche et de Formation sur le Paludisme (CNRFP), Ouagadougou, Burkina Faso; 7 Radboud Institute of Health Sciences, Radboud University Medical Center, Nijmegen, Netherlands

**Keywords:** malaria, treatment, pharmacology, mathematical modelling

## Abstract

**Introduction:**

Artemether-lumefantrine (AL) is the most widely-recommended treatment for uncomplicated *Plasmodium falciparum* malaria. Its efficacy has been extensively assessed in clinical trials. In routine healthcare settings, however, its effectiveness can be diminished by delayed access to treatment and poor adherence. As well as affecting clinical outcomes, these factors can lead to increased transmission, which is the focus of this study.

**Methods:**

We extend a within-host model of *P. falciparum* to include gametocytes, the parasite forms responsible for onward transmission. The model includes a pharmacokinetic–pharmacodynamic model of AL, calibrated against both immature and mature gametocytes using individual-level patient data, to estimate the impact that delayed access and imperfect adherence to treatment can have on onward transmission of the parasite to mosquitoes.

**Results:**

Using survey data from seven African countries to determine the time taken to acquire antimalarials following fever increased our estimates of mean total infectivity of a malaria episode by up to 1.5-fold, compared with patients treated after 24 hours. Realistic adherence behaviour, based on data from a monitored cohort in Tanzania, increased the contribution to transmission by 2.2 to 2.4-fold, compared with a perfectly-adherent cohort. This was driven largely by increased rates of treatment failure leading to chronic infection, rather than prolonged gametocytaemia in patients who have slower, but still successful, clearance of parasites after imperfect adherence to treatment. Our model estimated that the mean infectivity of untreated infections was 29–51 times higher than that of treated infections (assuming perfect drug adherence), underlining the importance of improving treatment coverage.

**Conclusion:**

Using mathematical modelling, we quantify how delayed treatment and non-adherent treatment can increase transmission compared with prompt effective treatment. We also highlight that transmission from the large proportion of infections which never receive treatment is substantially higher than those treated.

**Key questions**

**What is already known?**

- Artemether-lumefantrine (AL), the most widely-recommended treated for uncomplicated *Plasmodium falciparum* worldwide, has been proven in clinical trials to be efficacious and well-tolerated.
- In routine healthcare settings treatment efficacy can be diminished by delayed access to drugs, or imperfect adherence to the dosing regimen, although this effect is difficult to quantify.

**What are the new findings?**

- Using individual-level adherence data collected in Tanzania as input to our within-host model of a malaria infection treated with AL, we find that the onward transmission from a treated cohort of patients is more than double that of a perfectly-adherent cohort.
- In our model delayed access to treatment, determined from data on the time taken to acquire antimalarials following fever in malaria-endemic countries, increased onward transmission by up to 50%, compared with patients treated 24 hours after becoming febrile.

**What do the new findings imply?**

- We underline the importance of good adherence and prompt access to treatment, by showing how they can reduce onward transmission of the malaria parasite.
- Our modelling framework also allows us to compare the transmission capacity of treated patients with the much greater contribution to transmission made by untreated patients, emphasising the importance of extending treatment coverage.

## Introduction

Although significant declines in malaria prevalence have been achieved this century, recent evidence suggests that progress in reducing the burden of disease has stalled. In 2017 there were an estimated 219 million cases of malaria globally, compared with an estimated 217 million the year before.^1^ The key to bringing these numbers down is to increase access to vector control interventions, malaria diagnostic tools and quality-assured antimalarial treatment.^2^ Given the malaria parasite’s ability to develop resistance to antimalarial drugs,^3 4^ it is extremely important to maximise the efficacy of existing therapies, as well as identify novel antimalarial molecules to diversify the frontline therapies available to treat clinical disease. A number of factors contribute to suboptimal use of existing drugs, such as poor quality medication (including counterfeit medication), incorrect diagnosis of the cause of fever, a lack of access to WHO-recommended treatments, and poor adherence to the recommended treatment regimens.^5–10^ It is important to quantify how these factors reduce the levels of effective coverage in malaria endemic settings, and how this affects disease burden.^11 12^

Artemisinin-combination therapies (ACTs) are the frontline treatments for uncomplicated cases of *Plasmodium falciparum*, the human malaria species responsible for a large proportion of the global mortality. There are currently five WHO-approved ACTs available, all of which require a 3-day dosing regimen.^13^ The most widely-recommended ACT globally is artemether-lumefantrine (AL), which is the focus of our study. As of 2017, it is the first or second line treatment in 32 countries in Africa, 11 in the Americas, five in the Eastern Mediterranean, six in South-East Asia and five in the Western Pacific.^1^

In previous work we developed a within-host model of uncomplicated *P. falciparum* malaria in order to obtain an estimate of the impact of imperfect adherence to AL on treatment outcomes.^14^ To do this we used data on the number of doses taken and their timings from an unsupervised cohort in Tanzania collected in 2012.^15^ Our modelling work predicted a treatment failure rate of 4% if compliance was optimal (as in a clinical trial setting) but a higher failure rate of 9% if compliance was as observed. Of all the currently approved ACTs, AL has the most complex treatment regimen, requiring two doses per day instead of one, which may accentuate the difficulties in achieving good adherence.

Although the primary goal of an ACT is to clear the asexual parasite population and, therefore, restore the patient to health, artemisinin-based drugs also have a partial killing effect against the sexual-stage parasites (gametocytes), clearing developing gametocytes and reducing the circulation time of mature gametocytes. Both a female and male gametocyte, in their mature forms,^16^ must be taken up by a feeding *Anopheles* mosquito in order for onward transmission to occur. Treatment with an ACT can therefore reduce the probability that the patient will contribute to transmission after treatment. This is in contrast to other older treatments such as chloroquine and sulfadoxine-pyrimethamine, which have much weaker action against gametocytes.^17 18^

In *P. falciparum*, the delay between the appearance of asexual parasites and mature gametocytes in the peripheral blood is longer than in the other human malaria species.^19^ In a recent controlled infection study in malaria-naive human volunteers, it was measured to be 8.5–12 days.^20^ As artemisinin derivatives are highly effective against immature gametocytes,^21^ prompt treatment with an ACT can reduce the parasite’s transmission capacity. Delaying treatment allows more time for sexual-stage parasites to accumulate and mature, making an infected human more infectious to a feeding mosquito. For example, in a cohort of Nigerian children, a duration of clinical symptoms of more than 3 days was found to be a risk factor for having microscopy-detectable gametocytaemia at the time of presentation at a treatment facility.^22^

To assess the extent to which transmission increases due to delays to treatment and imperfect adherence, here we have extended our within-host model of asexual parasitaemia^14^ to also include sexual-stage parasites. We first modelled gametocytaemia in an untreated infection, and calibrated this model using data collected from neurosyphilis patients, who were infected with *P. falciparum* parasites to treat their syphilis infections.^23^ We have extended an existing pharmacokinetic–pharmacodynamic (PKPD) model for AL to quantify the effect on the sexual stage parasites, by fitting a PD model to individual-level clinical trial gametocyte density data following AL treatment. We estimated the probability that a mosquito would be successfully infected after taking a blood meal using a published model.^24^ We then combined these models with data on doses and timings collected from an unsupervised cohort in routine healthcare settings in Tanzania in 2012 to assess the contribution to transmission that stems from imperfect adherence; and we similarly used household survey data from endemic settings on the time taken to seek treatment to assess the role that delaying treatment can have on transmission. We also compared treated patients’ transmission capacity with untreated infections to assess the importance of improving treatment coverage in reducing malaria transmission at the population level.

## Methods

### Characterising the gametocyte dynamics

The modelling work developed for this analysis built on our previous published within-host model, which describes asexual parasite density over the course of an uncomplicated *P. falciparum* infection (details in Challenger *et al* 14). In that work, we described the dynamics of an untreated infection, including the immune response mounted against the parasite, using the malaria therapy dataset^23^ to calibrate the model. We used an existing population-PK model of AL,^25^ along with clinical trial data on AL efficacy, to calibrate a PD model of AL against asexual parasites. The timing of drug treatment in the model was guided by data on the time required to obtain antimalarials following fever in endemic settings^26^ and the observed pyrogenic thresholds of the malaria therapy patients.^27^ We here extended our investigation by including gametocyte production and characterising the effect that the two drugs have against the sexual-stage parasites. The drug model has been calibrated to data collected under controlled conditions that is, with perfect drug adherence. In the Results section, we explore the impact of imperfect adherence to the treatment regimen.

There are five morphologically-distinct gametocyte stages,^28^ which we label as $G_{1}$ (most immature) to $G_{5}$ (mature). Experimental evidence suggests that commitment to the sexual pathway is made at the schizont stage of the blood-stage cycle, placing all resulting merozoites on the gametocyte pathway.^29^ Immature gametocytes undergo sequestration, primarily in the bone marrow,^30^ returning to the blood circulation in their mature forms ($G_{5}$).

To characterise the relationship between a patient’s asexual and sexual parasitaemia, three factors need to be determined: the proportion of asexual parasites that commit to the sexual pathway, the length of time that gametocytes require to mature and the length of time that gametocytes (once mature) remain in the bloodstream. Using the malaria therapy dataset, detailed modelling work has been carried out to scrutinise these quantities, comparing models with a range of complexity.^31 32^ In these studies, large inter-individual variation was observed. The authors demonstrated that allowing the commitment rate to vary for each wave of asexual parasitaemia (see online supplementary methods) improved the model fit, that is, the proportion of parasites that develop into gametocytes can vary appreciably over the course of a single infection. Here we have built on these approaches, with several modifications. First, since our model of asexual parasitaemia has a time step of 48 hours, we generated gametocyte densities based on asexual parasitaemia data collected on odd days, instead of daily data. We explicitly modelled the sequestered parasite stages $\left(G_{1},G_{2},G_{3},G_{4}\right)$, to capture AL’s effect on both immature and mature gametocytes. For simplicity we assumed that the gametocytes spend an equal amount of time in each of the four immature stages. In vitro experiments do report the duration of the immature stages,^28 33–35^ but these values vary between studies. Finally, we model the gametocyte dynamics in continuous time, to ease the combination with the PKPD model of AL. The model equations can be found in the online supplementary methods.

10.1136/bmjgh-2019-001856.supp1 Supplementary data

The malaria therapy dataset contains daily measurements, stated as an integer number of parasites per µl, of both asexual and sexual parasitaemia for each patient, measured by microscopy.^23^ The full dataset contains infections from 334 patients: we fitted our model to a subset of 76, who did not receive any subcurative medication during the therapy and who were positive for gametocytes on at least 5 days. We use a patient’s asexual parasitaemia on odd days to predict that patient’s gametocytaemia, using a Poisson likelihood. The parameter combination that maximised the likelihood of the parameters for each patient was estimated using Markov Chain Monte Carlo methods. Further details can be found in the online supplementary methods.

### Modelling the impact of drug treatment on the gametocyte population

To describe the drug concentrations observed following treatment with AL, we used a population-PK model due to Hodel *et al*.^25^ The PK model was fitted to data from both children and adults, patients were provided with food and mothers were encouraged to breastfeed infant patients, as lumefantrine (LMF) is better absorbed with some fat. We also used this model to calibrate a PD model for AL against asexual parasites.^14^ In that work, we quantified how imperfect adherence can increase the probability that treatment with AL fails to clear the infection. We illustrate this in online supplementary figure 1, where we show how missing doses reduces drug concentration levels in the blood. Here we extended our PD model to include effects of AL against gametocytes. In order to reduce the parameter space, we assumed that artemether (AM) and its active metabolite dihydroartemisinin have identical antimalarial properties, and we ignored the effects of the active metabolite of LMF, desbutyl-lumefantrine, as its concentration is much lower than that of LMF. As stated previously,^14^ this means that the effect of the partner drug is only represented by the concentration of LMF. This may lead to the antimalarial properties of LMF being overestimated, but we do not expect this effect to be large. We also assumed that the two drugs act independently. Although our model contains all five gametocyte stages, it is not feasible to differentiate between the drug effect on for example, stages $G_{1}$ and $G_{2}$ as, in vivo, we only observe the mature, circulating gametocyte population. Furthermore, it is not straightforward to tease apart the relative contributions to gametocyte killing from AM versus LMF. The PD model has the form.

(1) $$\frac{dG_{i}\left(t\right)}{dt}=-k_{i}^{j}\left[\frac{C^{j}\left(t\right)}{C^{j}\left(t\right)+C_{50}^{j}}\right]G_{i}\left(t\right),\;i=\left(1,2,3,4,5\right),j=\left(AM,LMF\right)$$

Here $k_{i}^{j}$ denotes the maximum killing rate of drug j against gametocyte stage i and $C_{50}^{j}$ is the concentration at which the killing rate of drug j is half of its maximum value. We used in vitro data to guide the model fitting,^21^ specifically the relative strengths of gametocyte killing from AM versus LMF and the fact that the killing effects of both drugs are lower against more mature gametocytes. To reduce the parameter space, certain parameters were fixed during the model fitting process (see online supplementary methods).

To quantify the drug effect on gametocytes, we used data by Goncalves *et al*,^36^ in which asymptomatic children with patent gametocytaemia were treated with six doses of AL, quantifying gametocyte densities using quantitative reverse-transcriptase PCR. We compared our model output with gametocytaemia data for 42 patients, measured on day 0 (baseline), day 2, day 3 and day 7, while varying the PD parameters in the model. Full details of the model fitting are given in the online supplementary methods: briefly, we introduced a distance metric to describe the difference between patient data and simulated gametocytaemia in the model, as illustrated in online supplementary figure 2. We used likelihood-free inference methods^37^ to explore the PD parameter space.

### Translating gametocyte density into infectivity

We estimated the probability that a feeding mosquito would be successfully infected by using a recent study by Bradley *et al*,^24^ which analysed data collected from gametocyte carriers in Mali, Burkina Faso and Cameroon. The authors found that the best model for estimating the probability that a feeding mosquito would become infected used information on both the male and female gametocyte populations, rather than only the total gametocyte density. The authors also estimated a density-dependent relationship between the male and female gametocyte densities in each infection, which shows a bias towards male gametocytes at low gametocyte densities and a bias towards female gametocytes at higher densities. As our model describes how the total gametocyte population changes over time, we used the relationship for the sex ratio to estimate the male and female gametocyte densities, and translated this into an infectivity using the published model.^24^ This procedure is described in the online supplementary methods.

### Survey data for times to obtain treatment

To investigate the potential effect of delay to treatment in endemic settings, we used data from cross-sectional household surveys in subSaharan Africa in 2016 and 2017 and in which caregivers were asked to report the time taken to obtain antimalarial medication following fevers in children under 5 years old.^26^ Here, we further stratified the data by country, fitting a separate log-normal distribution in each case (see online supplementary figure 3 for the data and distributions). Data from seven countries were used: Burundi, Ghana, Sierra Leone, Ethiopia, Malawi and Uganda.

### Patient and public involvement

There was no patient or public involvement in the development or execution of this research study.

## Results

### Gametocyte dynamics in untreated and treated infections

**Figure 1:**
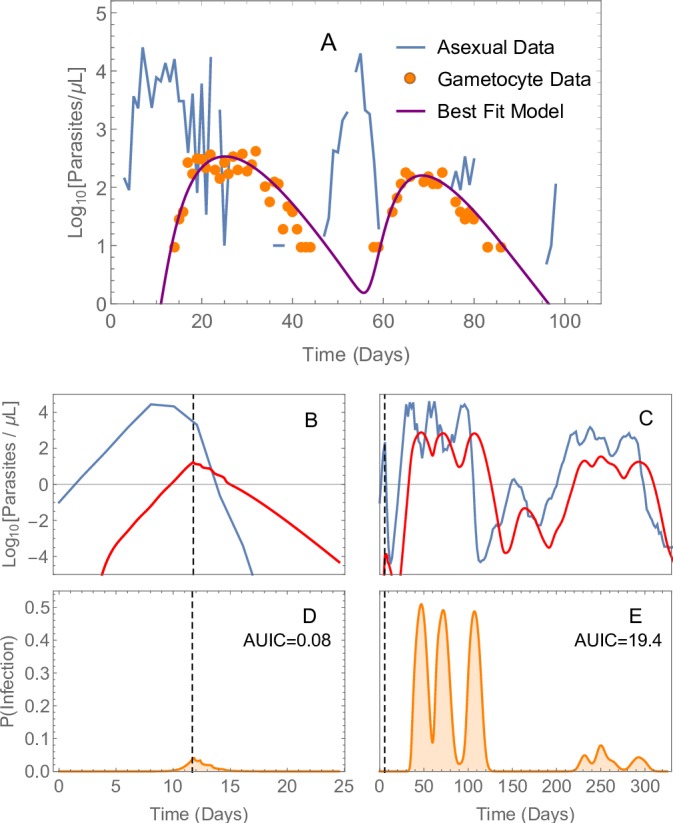
Panel (A) displays malaria therapy data for one patient, showing asexual (blue line) and sexual (orange circles) parasitaemia. the purple curve shows the best-fit model for gametocyte density. Based on the patient’s asexual parasitaemia, we estimated parameter values determining the proportion of parasites committing to the sexual pathway, the sequestration time of immature gametocytes and the circulation time for mature gametocytes. For this patient, the best-fit model had a maturation time for gametocytes of 9.8 days (parameter β=59.1 hours), and a mean circulation time for mature gametocytes of 9.2 days (parameter $\beta_{5}=221.3$ hours). The commitment rate during the first wave of asexual parasitaemia was $\alpha_{1}=0.021$. Panels (B)–(E) show two illustrative model simulations where fully adherent, prompt treatment with artemether-lumefantrine either successfully clears parasitaemia (panel (B)) or, alternatively, the parasitaemia rebounds (panel (C)). The vertical dashed line indicates the timing of the first dose of treatment. The blue lines in panels (B) and (C) show the density of asexual parasites and the red lines show the density of gametocytes. Panels (D) and (E) indicate the infectiousness over time of the two simulated patients, based on their gametocytaemias. Here P(Infection), the probability that a feeding mosquito is infected, is calculated on each day of the malaria episode. To quantity the total infectiousness of an episode of malaria, we calculated the area under the infectivity curve (AUIC), the value of which is displayed on the panel. For the case in which treatment failed, the patient did not receive a second course of antimalarials in this instance.

Gametocytaemia in untreated infections is determined by the fraction of asexual parasites committing to the sexual pathway, the maturation time for gametocytes and the circulation time of the mature gametocytes (see also the Methods section and online supplementary methods). The observed inter-individual variation, which is very high for all three parameters, is captured by the distributions defined in table 1. For example, the mean value for the circulation time of mature gametocytes was 7.7 days while for some patients a circulation time of twice this value was required to provide a good model fit. The median value for the sexual commitment rate, α, was 0.011. This is higher than the value found for another model that use this dataset^32^ as we used asexual parasitaemia on odd days, rather than daily data, to generate the observed gametocyte densities. The mean maturation time was found to be 7.6 days (a duration of 1.9 days in each immature gametocyte stage). The best-fit model for one patient is shown in figure 1.

**Table 1:** Part A: summary of parameter values describing gametocytaemia in untreated infections, as fitted to 76 malaria therapy patients. Best-fit values were obtained for each patient, as described in the Methods section. The distributions stated here describe the large variation observed within the group of patients studied. In each case, four distributions were assessed for goodness of fit (normal, log-normal, gamma and Weibull distributions). We sample from these distributions to generate dynamics in our within-host model. The median value of α is 0.011, while the mean gametocyte maturation time is 7.6 days. Part B: best-fit parameters and units for the PD model, describing the rate at which AL kills gametocytes, as defined in Eq. (1). Parameter $k_{i}^{j}$ is the maximum (max.) killing rate for drug j against gametocyte stage $G_{i}$. Eq. (1) shows how killing rates depend on drug concentration, with killing rates halved at the $C_{50}$ concentration of each drug. For both drugs we retain the $C_{50}$ concentrations fitted in a previous publication.^14^

A. Parameters governing the gametocyte dynamics		
Parameter	Distribution	Description
α	Log-normal distribution μ=−4.4, σ=1.9. Distribution truncated at 0.3	Commitment rate of asexual parasites
1/β	Normal distribution, μ=1.9, σ=0.6. Distribution truncated at (1, 3)	Mean time spent in each immature gametocyte stage (in days)
$1/\beta_{5}$	Normal distribution, μ=7.7, σ=4.1. Distribution truncated at (1.4,16)	Mean circulation time of mature gametocytes (in days)

B. Pharmacodynamic parameters for AL against gametocytes				
	Value for AM (95% credible intervals in brackets)	Value for LMF	Fixed or Fitted	
$k_{1}^{j}$	0.18	0.13	Fixed	Max. killing rate of stage 1 and 2 gametocytes (1/hour)
$k_{3}^{j}$	0.11 (0.09–0.12)	0.04	Fitted for AM, fixed for LMF	Max. killing rate of stage 3 and 4 gametocytes (1/hour)
$k_{5}^{j}$	0.08 (0.07–0.10)	0	Fitted for AM, fixed for LMF	Max. killing rate of stage 5 (mature) gametocytes (1/hour)
$C_{50}^{j}$	3.3	125	Fitted previously for asexual parasites^14^	Drug concentration (ng/ml) at which killing rate is half of its maximum

AL, artemether-lumefantrine; AM, artemether; LMF, lumefantrine.

We next used clinical trial data from Burkina Faso^36^ to characterise the drug action of AL against the sexual stage parasites (see the Methods section and online supplementary methods for full details). Asymptomatic children with patent gametocytes at screening were treated with AL and were followed up to track their gametocyte carriage over the subsequent days. The best-fit PD parameters that define Eq. (1) in the Methods section are given in table 1, which also specifies which parameters were fitted and which were held fixed. It is important to note that the PD model is intimately coupled to the PK model and should be viewed in that light. We found that the best-fit model required that the artemisinin-derivative have some effect against mature gametocytes, unlike the model in Gerardin *et al* 38 although this effect is much weaker than our model estimate against asexual parasites.^14^

### Running the model and estimating the infectivity of a malaria episode

Our within-host model is summarised in online supplementary figure 4, the caption of which lists the data sources used for calibration. In the model, approximately 5% of perfectly-adherent patients experience parasitological treatment failure, as found in clinical trials (see eg Atwine *et al* 39). Panels (B) and (C) of figure 1 shows two illustrative simulations: one where AL successfully clears the infection, and another where treatment fails and the asexual parasitaemia rebounds, leading to renewed production of gametocytes. As described in the online supplementary methods, we used the gametocyte density to estimate the patients’ infectivity using the model due to Bradley *et al*.^24^ To quantify a patient’s total infectivity over the course of an infection, we calculated the area under the infectivity curve (AUIC) (yellow area in panels (D) and (E) of figure 1). In the case where treatment succeeds, the clearance of the asexual parasitaemia prevents further production of gametocytes; in this instance the AUIC was 0.08. This means that, if the patient was bitten once a day for the duration of the infection they would, on average, infect 0.08 mosquitoes. The simulation in which treatment fails demonstrates the large effect that treatment failure has on the total infectiousness of a malaria infection. Since, in this example, the recrudescent infection is not retreated, its total infectivity is comparable to a completely untreated infection (in this case the AUIC was 19.4).

These simulations suggest that an important contribution to onward transmission can stem from treatment failure if the recrudescent infection is not retreated. This depends on whether symptoms return, as well as whether a person seeks a second course of treatment. We reviewed nine clinical trials that stratified day 28 PCR-corrected failure rates into late clinical failures versus late parasitological failures (LPF) that is, the latter have no symptoms when parasites recur. Overall, 12 out of 46 patients (26.0%) were classified as clinical failures.^40–48^ This percentage may vary with the level of naturally acquired immunity but assessing this was beyond the scope of this investigation. Furthermore, the majority of studies considered did not have data on the likelihood of an LPF progressing to a symptomatic failure after day 28. Throughout this study, we varied the proportion of treatment failures that receive a second course of medication, to show how this affects the results obtained. In the case where a recrudescent infection is retreated, the second course of treatment is sought after the patient’s parasitaemia exceeds their pyrogenic threshold.

### The impact that delayed treatment has on transmission

**Figure 2:**
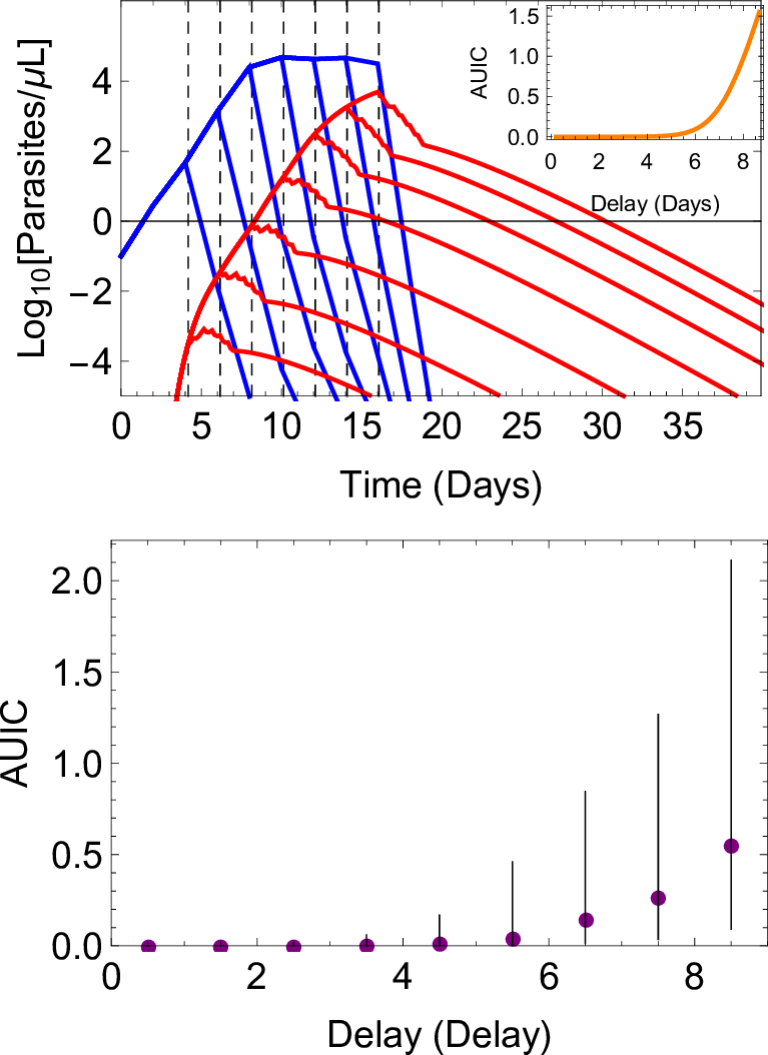
Simulation results illustrating how increasing the time taken to obtain treatment following the onset of fever leads to increased infectivity. the upper panel shows a simulated infection, with asexual parasite density in blue and gametocyte density in red. Seven model runs are shown, varying the time at which treatment with artemether-lumefantrine starts (vertical, dashed lines) and removing variation due to other factors (eg, individual-level variation in the PK model, the patient’s pyrogenic threshold, maturation and circulation times of the gametocytes, etc). For each model run, the area under the infectivity curve (AUIC) was calculated. The relation between the AUIC and delay to treatment in this instance is shown in the inset. More general results are shown in the lower panel, where results were obtained from a simulated cohort of 10 000 patients, to highlight the individual-level variation in the model. Delay to treatment was grouped into 1-day-wide bins. for each bin, we summarise the infectivity of the episodes by showing the median AUIC (filled circles) and the interquartile range (vertical lines). for all results presented here, adherence to treatment was perfect.

In the model, the timing of treatment is determined by the parasite density at which fever commences (the pyrogenic threshold), and the subsequent time taken to seek treatment.^14^ Delaying access to treatment allows the build-up of the gametocyte population and, therefore, increases the capacity for transmission, even when treatment is subsequently successful. We illustrate this effect in figure 2, both at the individual and cohort level, showing how changing the time to obtain treatment affects the size of the gametocyte population. Generating a large ensemble of simulations, we show that infections treated within 24 hours of the development of symptoms are highly unlikely to contribute to transmission (median AUIC=0.0019), whereas infections treated after a delay of a week are more likely to contribute to transmission (median AUIC of 0.27).

**Figure 3:**
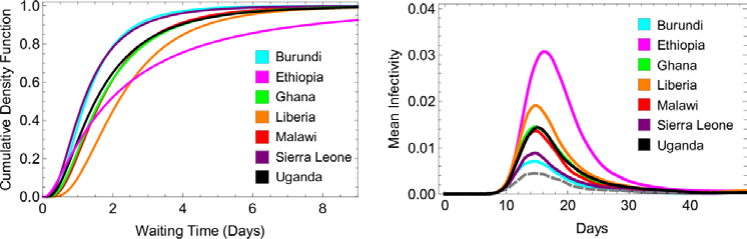
Model fits to data describing delay to obtaining antimalarial treatment in seven sub-Saharan African countries, and model estimates for how this affects infectivity to mosquitoes. The left panel shows the best-fit log-normal distribution in each country for time taken to obtain antimalarial medication after the onset of fever. The raw data for each country is displayed in online supplementary figure 3. To determine the timing of the first dose of artemether-lumefantrine in our within-host model, we sampled from one of these distributions. The right panel shows how each distribution affects the model-estimated infectivity of a treated malaria infection over time, focussing on the first wave of gametocytaemia. For each distribution, we averaged results over 10 000 simulations. According to the model and survey data, mean infectivity is lowest when the data collected in Burundi and Sierra Leone is used (turquoise and purple, with mean AUICs of 0.52 and 0.53 respectively), and highest for Ethiopia (pink, mean AUIC of 0.71). The AUICs shown here pertain to the case where all recrudescent infections are retreated. this panel also shows the average infectivity when treatment is obtained 24 hours after the onset of fever, reflecting WHO treatment guidelines (grey dashed line, mean AUIC 0.47). AUIC, area under the infectivity curve.

To investigate the potential effect of delay to treatment in endemic settings, we used data from cross-sectional household surveys in sub-Saharan Africa in 2016 and 2017 and in which caregivers were asked to report the time taken to obtain antimalarial medication following fevers in children under 5 years old^26^ In the seven countries considered, treatment was obtained most promptly in Burundi and Sierra Leone, where 72% of patients reported receiving treatment either the same or next day after fever onset. In Ethiopia, 45% of patients reported receiving treatment either the same or next day after fever onset, while 20% reported that four or more days were required to obtain treatment (compared with only 2% in Burundi and 1% in Uganda). In figure 3 we show how the time to obtain treatment impacts the mean infectivity of a simulated cohort of patients in each country, compared with a cohort who receive treatment after 24 hours, in line with WHO recommended policy.^13^ With the times to treatment reported in Ethiopia, we estimate that the mean infectivity of a treated malaria episode is 1.5 times greater than when treatment is received after 24 hours, assuming all recrudescent infections were retreated. This effect is due to a greater contribution to infectivity from the first wave of gametocytaemia, driven by the proportion of the cohort waiting several days for treatment.

### The impact that imperfect adherence has on transmission

**Figure 4:**
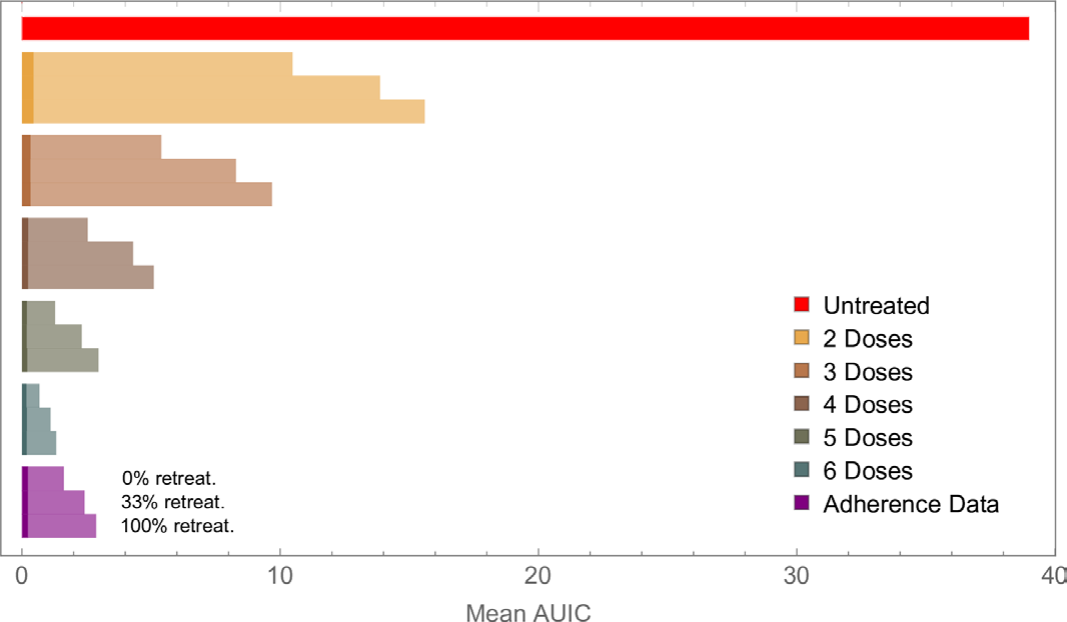
Model output showing how failure to complete treatment can increase onwards transmission of the parasite. Results for perfectly-adherent patients (six doses) are compared with those for patients who did not take all doses. The bars marked ‘five doses’ shows results for patients who took only the first five doses, the bars marked ‘four doses’ shows results for patients who took only the first four doses, and so on. We also compare results for perfectly-adherent patients with those obtained using data on adherence to artemether-lumefantrine, collected in Tanzania in 2012 (purple bars). In each case, we examined three scenarios for retreating recrudescent infections, as indicated for the three lowest bars. In each bar, the lighter colour shows infectivity due to treatment failure in the cohort, while the darker shade represents infectivity from gametocytes that circulate after treatment. this latter contribution to the mean infectivity (mean AUIC) was 0.19 for the perfectly-adherent cohort, compared with 0.23 for the cohort simulated with the adherence data, and 0.43 for the cohort that took only the first two doses. For the results generated from the Tanzanian cohort, the mean AUIC was calculated from an ensemble of 1000 simulations for each of the 482 adherence profiles. In the other scenarios, the mean AUIC was calculated from 10 000 model simulations. For comparison, the mean AUIC for a completely untreated cohort was 39.0 (red bar). Hence for perfect drug adherence, treated infections were between 29 (no recrudescent infections retreated) and 51 (all recrudescent infections retreated) times less infectious than untreated ones. All results shown here used the delay-to-treatment distribution fitted to demographic health survey data from all available sub-Saharan African countries, which was used in our previous work.^14^ AUIC, area under the infectivity curve.

Poor adherence to a treatment regimen slows the clearance of gametocytes after treatment, as well as increasing the probability that treatment fails to clear the asexual parasite population. We estimated mean infectivity for patients who completed treatment with those who missed doses, starting by removing the sixth dose, then removing the fifth and sixth dose and so on (figure 4). In each case, three scenarios for retreatment were used: (i) No patients receive a second course of medication; (ii) Patients who fail treatment receive a second course of AL with probability 1/3; (iii) All patients who fail treatment receive a second course of AL. For each scenario we examine the relative contributions from residual gametocytes post treatment (which all patients will have, regardless of whether treatment is successful), and gametocytes produced following treatment failure. In the case of perfectly-adherent patients, retreating all recrudescent infections nearly halves the mean AUIC for the cohort, reducing it from 1.32 to 0.67 (figure 4). These results indicate that, although only a small percentage (~5%) of adherent patients fail treatment, these individuals may make relatively large contributions to the mean infectivity of the cohort.

We observe large increases in average infectivity when the treatment regimen is not completed (figure 4). For example, in the case where no recrudescent infections are retreated, our model suggests that patients who missed the last dose out of the total of six were 2.2 times as infectious as fully-adherent patients, while patients who missed the last two doses were 3.8 times as infectious. Although a small part of this effect is due to slower-clearance of gametocytes after treatment (under 1%, for both patients who took five and four doses), the increases in infectivity are driven almost entirely by increased treatment failure rates.

We further estimated the impact of imperfect adherence using individual-level data collected in Tanzania in 2012.^15^ Here, AL was dispensed in Smart Blister Packs, which recorded the timing of when each pill was removed from the packet in patients who were largely unsupervised, except sometimes for the first dose. As per Challenger *et al* 14 we used data from 482 patients as input for our model, and compared the mean infectivity obtained with those found for perfectly-adherent patients, again exploring three retreatment scenarios for recrudescent infections. In the case where one-third of patients were retreated following treatment failure, the observed adherence patterns increased mean infectivity by a factor of 2.2 compared with perfectly-adherent patients (figure 4). When no patients were retreated, mean infectivity increased by a factor of 2.2, and in the case where all patients were retreated mean infectivity increased by a factor of 2.4. The variation in these values can be explained by noting that the total infectivity of a cohort stems from two sources: residual gametocytes that circulate following treatment, which are present regardless of whether the infection is successfully cleared, and gametocytes produced following treatment failure (see figure 4). Only the contribution from the latter can be reduced by retreating recrudescent infections. Therefore, the proportion of total infectivity that is due to first-wave gametocytes increases when more recrudescent infections are treated. Again, the observed increase in average infectivity is driven largely by additional treatment failures, although we also observe a small contribution from post treatment gametocytaemia (about 6% of the increase in mean infectivity for the cohort that received no retreatment).

## Discussion

The efficacy of malaria treatment and its impact on transmission potential are typically measured under controlled conditions, rather than in routine healthcare settings. Here, we estimated that onward transmission from a treated cohort of patients with realistic adherence patterns is more than double that of perfectly-adherent patients. The major driver of this increased transmission in our analysis is treatment failure without retreatment, leading to chronic subsequent infections of long duration. Imperfect adherence also led to slower clearance of gametocytes following treatment, regardless of whether the asexual parasites were successfully cleared, but this had a relatively small impact on transmission potential.

We also looked at how delays in obtaining antimalarials following fever affects onward transmission, based on data from seven endemic countries. Assuming patients were fully-adherent, we found that using these waiting times in the model increased patients’ capacity to transmit malaria by up to 1.5-fold, compared with patients treated after 24 hours. This effect stems from the long maturation time of gametocytes in falciparum malaria, and the high susceptibility of immature gametocytes to artemisinin derivatives. Our analysis quantifies the role that prompt and effective treatment has in reducing onward transmission, and of course it also has a critical role in alleviating clinical symptoms and reducing the risk of severe disease which we did not quantify here. A limited amount of data was available for some countries, such as Ethiopia and Malawi, and timing of treatment is self-reported. Therefore, the data should be interpreted as illustrative of the range of current access times, rather than painting a detailed picture of access to antimalarials in the countries considered. Furthermore, access to treatment within a country is likely to be highly heterogeneous and change over time. As more data becomes available, more detailed models for this variable can be developed, rather than assuming a single distribution for each country.

While timing and adherence are important within cohorts of treated patients, our analysis suggests that in situations where a significant proportion of infections remain completely untreated, the contribution to transmission from these infections may be much greater than contributions from treated infections. This has been found to be the case in a number of modelling studies.^38 49–52^ In our model, for example, the mean AUIC from an untreated symptomatic cohort is 35 times higher than that of a cohort with realistic adherence patterns (assuming one third of infections are retreated, see figure 4). This is particularly relevant considering evidence that in sub-Saharan Africa in 2015 only 19.7% of symptomatic RDT-positive children under 5 years old in sub-Saharan Africa received an ACT.^10^ Therefore, increasing access to treatment remains a priority. Efforts in this direction are being made, both to improve affordability of quality-assured ACTs, and their availability in remote areas.^53 54^ Expanding access to treatment can be implemented by community-health workers, as recently reported in Rwanda.^55^ This type of approach can also reduce the time required to obtain treatment, as the need to travel to a health centre is removed.

Some published within-host models, largely informed by malaria therapy data, contain both asexual and sexual parasites, and consider effects of antimalarial drugs on both parasite populations.^38 50^ Our approach is broadly similar, although our PD model against gametocytes is calibrated against clinical trial data at the level of individual patients, rather than at a cohort level. To our knowledge, this approach has not been carried out before. Furthermore, we believe that the combination of a within-host modelling framework with individual-level adherence data provides much-needed insight into the impact of poor adherence at both the patient and population level. It is instructive to compare our estimates for the impact of treatment on infectivity to those obtained by Johnston *et al*.^50^ In that study, the authors explore a range of treatment scenarios and report the ‘effect size’ of treatment, defined as the fold reduction in infectivity due to treatment. Here the authors used an infectivity model due to Jeffery and Eyles,^56^ from a study of mosquito feeding on malaria therapy patients. For treatment with an ACT, the effect size of treatment was estimated to be 87.3, much higher than our range of 29–51 (see figure 4), and up to 162.1 with the addition of primaquine (see table 2 of Johnston *et al* 50). The main reason our treatment effect size appears much smaller is that treatment is not always successful in our model: even when adherence is perfect infectivity from a treated cohort is boosted due to the 5% of infections that recrudesce. The choice of infectivity model also influences the numerical results obtained. Johnston *et al* also used a second infectivity model, due to Carter and Graves^57^ and based on data from multiple studies, which estimated much smaller effect sizes for treatment (less than 10). Such different results are obtained because the two models generate very different estimates for an individual’s infectivity prior to treatment. We believe that using a recently-published infectivity model,^24^ which quantified gametocyte densities using quantitative reverse transcriptase PCR rather than microscopy, enables us to make an improved assessment of infectivity, based on an individual’s gametocytaemia.

Although we have only considered treatment with AL in this work, the framework outlined here would enable similar PD models to be fitted for other ACTs given appropriate data. In addition, interventions that are designed to target mature gametocytes rather than the asexual parasites, such as single low-dose primaquine, could also be assessed. To do this, however, the model would need to reflect the sterilising effect of some gametocytocidal drugs precedes the gametocyte clearance effects.^58 59^ In this work, we have considered all circulating gametocytes to be fully viable. A more detailed model, assessing the sterilising effects of antimalarial drugs, would be a very interesting avenue for further work.

There are several limitations to our analysis. Adherence and treatment-seeking behaviour may vary with age, which we have not explored here. There is evidence that adults receive more mosquito bites than young children, which would influence their relative transmissibility.^60^ As discussed in the Methods section and online supplementary methods, more detailed parasitaemia models would provide more precise quantitative insight into the process of gametocytogenesis. Also, our model of parasitaemia in untreated infections is calibrated against data from infected individuals with little or no previous exposure to malaria. We do not consider the role of immunity acquired by exposure over a longer time period, which will reduce the parasite densities of malaria infections, as well as reducing the proportion of infections that become symptomatic. Therefore, our results are most relevant in low-transmission settings, particularly as it is likely that a lower proportion of infections go untreated, increasing the importance of treatment in reducing transmission. It is in precisely these settings where one would hope to interrupt transmission using a range of tools, including good access to treatment.

Incorporating naturally-acquired immunity into well-calibrated within-host malaria models remains a challenging undertaking, due to the lack of longitudinal data from untreated infections. One model used cross-sectional data from malaria endemic areas to assess how acquired immunity reduces parasite densities in infections.^61^ The results presented here will also vary depending on the duration of untreated falciparum infections, which display large variation between patients,^62^ and will vary with exposure to the parasite.^63 64^

## Conclusions

We have used within-host modelling to show how delays in obtaining antimalarials and imperfect adherence to the dosing regimen can lead to increased transmission of the parasite. These effects are not readily measurable in clinical trial settings for clear ethical reasons. As well as quantifying how delayed treatment and imperfect adherence to the dosing regimen can increase a treated individual’s capacity to transmit malaria, our work suggests that the major priority should be reaching patients who do not currently have access to treatment. The WHO’s Global Technical Strategy for Malaria aims to reduce the global malaria burden by 90% by 2030,^2^ and aiming to achieve universal access to quality-assured antimalarials is one of the key tools in helping to achieve this.
