# Supplementary material for: How delayed and non-adherent treatment contribute to onward transmission of malaria: a modelling study
Source: BMJ Glob Health. 2019 Dec 10;4(6):e001856. doi: 10.1136/bmjgh-2019-001856 (PMC6936434; doi:10.1136/bmjgh-2019-001856)

Supplementary Materials

Supplementary Methods

Gametocyte dynamics in untreated infections

A differential equation model describes the dynamics of the gametocyte population. We assume the time spent in each stage is gamma distributed, to provide a good visual match to *in vitro* data, splitting each stage into L auxiliary compartments. We selected the simplest model that provided a reasonable visible match to the *in vitro* data ($L = 3$). We write the total population of circulating gametocytes as $G_5(t) = G_5^1(t) + G_5^2(t) + G_5^3(t)$. The equations describing the gametocyte dynamics are:

$$\begin{aligned}
\frac{dG_k^1}{dt} &= (1-\delta_{1k})\gamma_{k-1}G_{k-1}^3 - \gamma_k G_k^1, \\
\frac{dG_k^2}{dt} &= \gamma_k G_k^1 - \gamma_k G_k^2, \\
\frac{dG_k^3}{dt} &= \gamma_k G_k^2 - \gamma_k G_k^3, \quad k = (1,2,3,4,5).
\end{aligned} \qquad \text{(S1)}$$

To tidy the notation we introduce $\gamma_k = (\beta L, \beta L, \beta L, \beta L, \beta_5 L)$, where $1/\beta$ is the mean residency time in each immature gametocyte stage, and $1/\beta_5$ is the mean circulation time of the mature gametocytes. The Kronecker Delta ($\delta_{ij} = 1$ if $i = j$, $\delta_{ij} = 0$ if $i \neq j$) simply indicates that input to the first gametocyte compartment is not included in the differential equation model. Instead, every 48 hours this compartment receives an influx of newly-committed gametocytes from the asexual parasite population, governed by our previously published model (14). A more detailed, continuous time model of asexual parasitaemia would have a flow of newly-committed gametocytes into G_1^1, caused by the rupture of erythrocytes.

The full malaria therapy dataset contains 334 patients: we focused on 89 patients, who did not receive any sub-curative medication during the therapy. We then removed patients who had detectable gametocytaemia on four days or fewer, as it was not possible to estimate the circulation time of mature gametocytes in these cases. This left us with data from 76 patients.

Following previous studies (31,32), we used only data from the first 100 days of an infection. Both the gametocyte maturation time and circulation time of mature gametocytes were assumed to be constant throughout an individual infection. The sexual commitment rate (α) was allowed to vary for each associated wave of asexual parasitaemia. In Refs. (31,32), the authors defined waves of parasitaemia by identifying local maxima of parasite density, which are defined as having a greater parasite density than the six preceding measurements and a greater or equal parasite density than the following six measurements. In this way, the infection can be segmented into successive waves of parasitaemia. The authors speculated that there could be a link between sexual commitment rate and the expression of different variants of protein PfEMP1, which plays an important role in both the sequestration of asexual parasites, and the antigenic variation observed over the course of an infection (Ref. 32). However, there is evidence that the commitment rate can alter in response to changes that occur in the host during the course of the infection and that this is responsible for the

time-varying commitment rates (see e.g. Reece et al. Proc R Soc Lond B 272, 511-517 [2005] or Carter et al. Evol Med Public Health 135-147 [2013]). As the number of waves of asexual parasitaemia is not the same for all patients, we fitted the model to each malaria therapy patient separately. We use a patient's asexual parasitaemia on odd days to predict that patient's gametocytaemia, using a Poisson likelihood. The parameter combination that maximises the likelihood of the parameters for each patient was estimated using Markov Chain Monte Carlo methods. After this procedure was carried out for all 76 patients, we fitted distributions to these best-fit parameters, assessing a range of two-parameter distributions (Table 1). We sample from these distributions to simulate gametocyte dynamics in our within-host model. We note that, as our asexual parasitaemia has a 48-hour time-step, we observe higher values for the sexual commitment parameter than found in models that use daily data on asexual parasitaemia. This means that our parameter values have less biological relevancy.

For the results presented in this article, we fixed the sexual commitment rate for each individual model run, even though it was allowed to vary for each wave of asexual parasitaemia during the model fitting process. This choice simplifies the running of the model: if sexual commitment varies during an infection, the model for asexual parasitaemia must be run *first*, to assess the number of waves of asexual parasitaemia, as well as their timings and durations. Only afterwards can one run the full model, containing both asexual and sexual parasitaemia. In treated infections, the first wave of parasitaemia is generally the most significant (if treatment is successful). Furthermore, our primary interest is at the cohort level, averaging over many patients, rather than attempting to capture all the individual-level variation observed during an infection of *P. falciparum*.

Calibrating the pharmacodynamic model

The set of equations governing the PD model is Eq. 1 in the main text. To reduce the parameter space, we assume that the C_{50} values determined previously for our model of asexual parasitaemia (14) are valid here, so that only parameters k_i^j need to be determined. We also assume that the effect of each drug is the same against stage 1 and 2 gametocytes, setting $k_1^j = k_2^j$, and the same against stage 3 and 4 gametocytes ($k_3^j = k_4^j$), where $j = (AM, LMF)$. Observations from *in vitro* data suggest that the effect of lumefantrine on mature gametocytes is small (21), and so we set $k_5^{LMF} = 0$.

In vitro studies clearly show that the killing effect of both drugs is stronger in young gametocytes (21). Therefore, we set the values of parameters k_1^{AM} and k_1^{LMF} to be similar to those fitted for asexual parasites (14). We fixed the value of k_3^{LMF}, giving lumefantrine a small killing effect against stage 3 and 4 gametocytes (21), and allow k_3^{AM} to vary in the model fitting. With this parameter space reduction we are left with two PD parameters which must be fitted to the dataset, k_3^{AM} and k_5^{AM}. We assume that the relative magnitudes of the killing effects in the *in vitro* experiments will be consistent with those found *in vivo*. Therefore, we select a flat prior in the model fitting, but add the constraints that $k_1^{AM} > k_3^{AM} > k_5^{AM}$.

To quantify the drug effect on gametocytes, we use data for patients with patent gametocytaemia at the commencement of treatment and follow the gametocyte density over the subsequent days. We utilise data by Goncalves et al. (36) who treated asymptomatic children with patent gametocytaemia

with six doses of AL, quantifying gametocyte densities using quantitative reverse-transcriptase PCR. We compared our model output with gametocytaemia data for 42 patients, measured on day 0 (baseline), day 2, day 3 and day 7, whilst varying the PD parameters in the model. For patient i we denote this data as $H^i = (H_0^i, H_2^i, H_3^i, H_7^i)$.

Within our model, a patient's gametocytaemia at the start of treatment is determined by α, β, and β_5 (the parameters fitted from the untreated infections), as well as the recent asexual parasite density. Therefore, baseline gametocytaemia varies greatly in a simulated cohort of patients (as illustrated in Supplementary Figure 2) and, due to the model's intractability, cannot be pre-selected by the user.

For a given baseline gametocyte density, various factors in the model generate variation in the gametocytaemia in the days after treatment starts. These include: variation in pharmacokinetics, circulation time of mature gametocytes, and the size of the sequestered gametocyte population. To fit the PD model, we define a distance function $\rho(G, H^i)$ to describe the similarity of a simulated infection (G) with the gametocyte data for each patient (H^i). In the model, the density of mature gametocytes is $G_5(t) = G_5^1(t) + G_5^2(t) + G_5^3(t)$, summing over the auxiliary compartments. We denote the time in the model at which treatment starts as t_l and define $\rho(G, H^i)$ as

$$\begin{aligned}\rho(G,H)^2 = &(\log_{10} G_5(t_l) - \log_{10} H_0^i)^2 + (\log_{10} G_5(t_l+2) - \log_{10} H_2^i)^2 \\ &+(\log_{10} G_5(t_l+3) - \log_{10} H_3^i)^2 + (\log_{10} G_5(t_l+7) - \log_{10} H_7^i)^2.\end{aligned} \quad \text{(S2)}$$

To fit the model, PD parameters are sampled from our prior distribution and we store parameter values that are consistent with $\rho(G, H^i) \le \varepsilon$, (see Algorithm C in Ref. (37)). For some patients, data for one or more days is missing, or the gametocyte density is recorded as zero, meaning that it is below the lower limit of detection (LLOD), which in this instance was 0.02 gametocytes per microlitre. In the first case we calculate Eq. 3 for the data points available, adjusting the value of ε accordingly, giving an equal importance to each of the four data points. When the gametocyte density for a patient is recorded as zero, we record the distance from the simulated trajectory to the LLOD. Or, if the simulated trajectory is also under the LLOD, that data point's contribution to $\rho(G, H^i)$ is set to zero.

In the limit $\varepsilon \to 0$, accepted parameter values are samples from the posterior distribution. In practice, of course, finite ε is required to find trajectories that satisfy the condition. We sought the PD parameter values for k_3^{AM} and k_5^{AM} that provide the best results obtained for the overall cohort. We did exclude one patient who was gametocyte-negative on days 2 and 3, but had detectable gametocytaemia on day 7. This is difficult to explain within the framework of the model, as day 7 is too early to consider mature gametocytes appearing from a recrudescent or new infection. One possibility is that some immature gametocytes survived three days of the artemisinin derivative and were then released into circulation. Removing this patient means that the best-fit parameters in Table 1 come from the remaining 41 patients.

Translating a gametocyte density into a probability of infecting a feeding mosquito

We have used a recently-published model of infectivity due to Bradley et al. (24). As mentioned in the Methods section, the authors of that study found that using both the male and female gametocyte densities, rather than the total gametocyte density, led to the best model for the probability that a feeding mosquito would become infected. The authors also estimated a density-dependent relationship between the male and female gametocyte densities measured in each individual. Here we utilised this relationship to partition the gametocyte density (say, z) predicted in our model into densities for male (x) and female (y) gametocytes. As shown Figure 1 of the original study (24), the authors fitted a power-law relationship (a straight line on a log-log plot) between the male and female gametocytes i.e.

$$\log_{10} y = a + b \log_{10} x .$$

In the original study, the parameter values obtained were: . $a = -0.20$ and $b = 0.67$. For a given z we can solve for x and y since we also know that $x + y = z$. Unfortunately, the simultaneous equations do not have a closed-form solution and must be solved numerically. For convenience, when running the simulation model in C++, a data table is provided for a large range of gametocyte densities. Hence, when calculating an infectivity the program accesses this pre-made table, rather than solving the simultaneous equations every time.

References

References are numbered as per the main text

Supplementary Figures

Supplementary Figure 1: Imperfect adherence and lumefantrine concentrations. This figure shows mirrored probability distributions of the lumefantrine concentrations 7 days after the start of treated, as predicted by the population-PK model due to Hodel et al. (25). For each adherence scenario, results were obtained from 500 model simulations. Some outliers have been discarded for clarity. In a previous study, we quantified the link between imperfect adherence and treatment failure (recrudescent of the asexual parasitaemia) using this PK model in harness with our PD model (14).

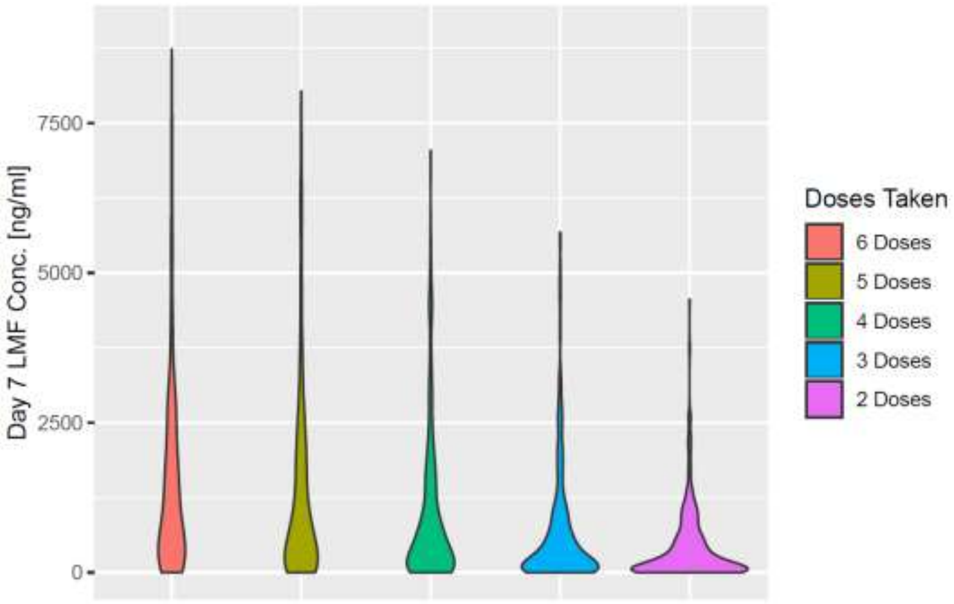

Supplementary Figure 2: An illustration of the procedure used to fit the PD model. In the upper panel we show an ensemble of model runs (red lines) for one choice of the PD parameters, along with the data (H^i) from one patient. For one trajectory, shown in purple, we calculate the distance metric between the data and the simulation output at the corresponding time points (G). For clarity we only show trajectories, for which the initial gametocyte density is within an order of magnitude of the data point at baseline. However, when performing the model fitting we consider all possible trajectories. In the lower panel, we repeat the procedure for another choice of the PD parameters. Here, we reduce the drugs' killing effect of gametocytes, in order to mimic a drug such as SP, which has a minimal killing effect on these parasites. This means that, following treatment, previously-sequestered parasites will boost the population of circulating parasites, in contrast to the behaviour found for ACTs. This means that the probability of a trajectory satisfying the condition $\rho(G, H^i) \leq \varepsilon$ for this patient is very low.

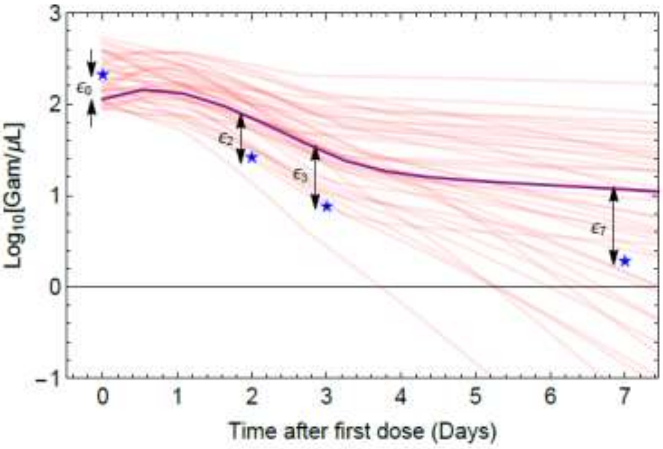

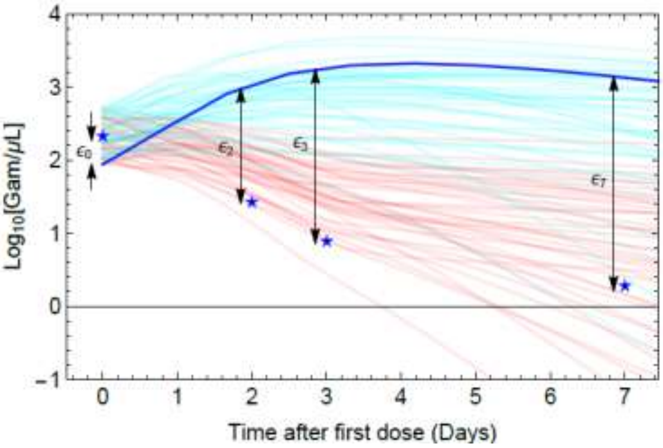

Supplementary Figure 3: Data collected from cross-sectional surveys on the time taken to obtain antimalarial drugs following fever. For each case, we fit a log-normal distribution, by calculating the maximum likelihood of binned data to the continuous distribution, as we did previously (14). These distributions were then sampled from to produce the results presented in Figure 3 in the main text.

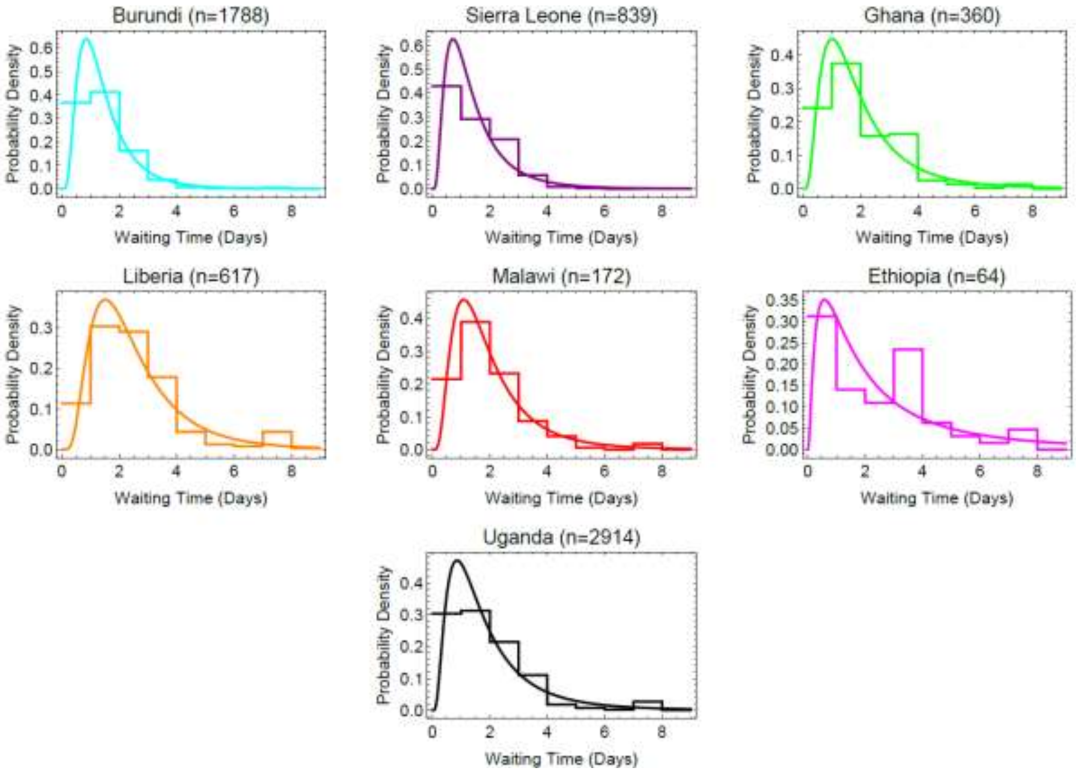

Supplementary Figure 4: Overview of the model, and the data used to inform it. After an infection commences, the parasite population expands and eventually exceeds the patient's pyrogenic threshold. If the infection is untreated, it can persist for a long time. If treatment is obtained, the time taken to acquire antimalarials is drawn from a distribution. The PKPD model determines the impact that treatment has on the infection, which will be strongly influenced by the adherence to the dosing regimen. If treatment fails and the infection recrudesces (which, even with perfect adherence, will happen 5% of the time), the infection can either be retreated or not. In the four scenarios shown in the diagram, we can calculate the patient's transmission capacity by calculating the area under the infectivity curve (AUIC). We now summarise the data sources that informed the model. [1]Malaria therapy data, described in Ref. (23), was used to fit the parasitaemia model and to inform the fever threshold (following work by Dietz et al. [27]). [2]Time required to obtain antimalarials fitted to data from Demographic Health Surveys (26). [3]Realistic adherence patterns comes from the Smart Blister Pack data, collected in Tanzania (15). [4]We used a published pharmacokinetic model (25) and calibrated the pharmacodynamic model of AL against gametocyte using work by Goncalves et al. (36). We calculated the pharmacodynamic model against asexual parasites previously (14). [5]To translate a gametocyte density into an infectivity we used a recently published model due to Bradley et al. (24). The size of the blue arrows indicates the relative capacity for transmission in each case (not precisely to scale).

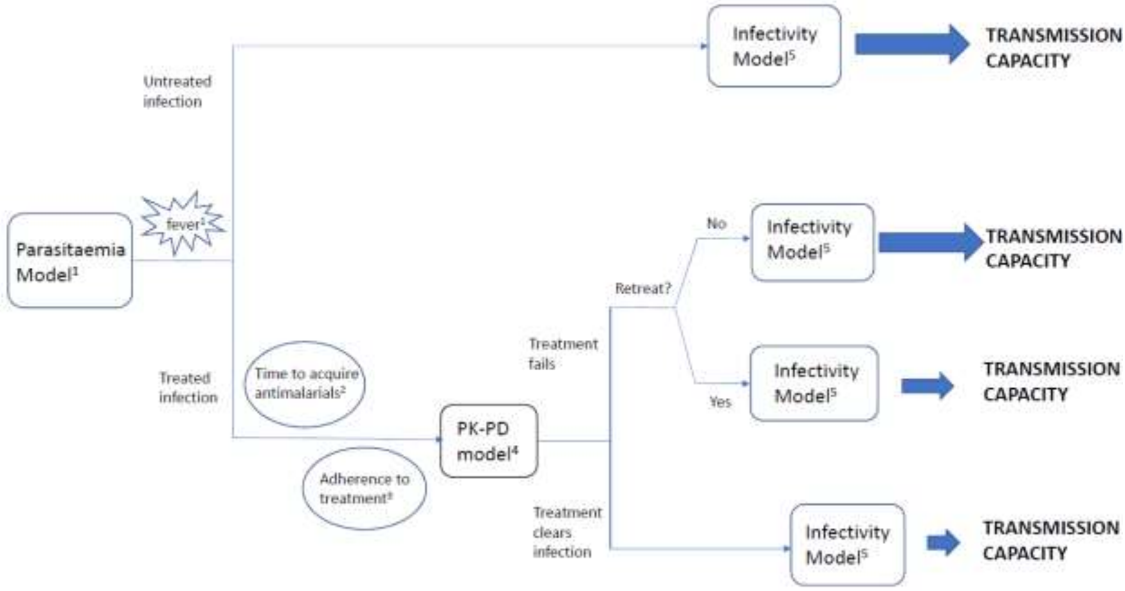

Supplement: Supplementary data [file bmjgh-2019-001856supp001.pdf]
